# Bacteria Endosymbiont, *Wolbachia*, Promotes Parasitism of Parasitoid Wasp *Asobara japonica*

**DOI:** 10.1371/journal.pone.0140914

**Published:** 2015-10-22

**Authors:** Shunsuke Furihata, Makiko Hirata, Hitoshi Matsumoto, Yoichi Hayakawa

**Affiliations:** Department of Applied Biological Sciences, Saga University, Saga, Japan; Alexander Fleming Biomedical Sciences Research Center, GREECE

## Abstract

*Wolbachia* is the most widespread endosymbiotic bacterium that manipulates reproduction of its arthropod hosts to enhance its own spread throughout host populations. Infection with *Wolbachia* causes complete parthenogenetic reproduction in many Hymenoptera, producing only female offspring. The mechanism of such reproductive manipulation by *Wolbachia* has been extensively studied. However, the effects of *Wolbachia* symbiosis on behavioral traits of the hosts are scarcely investigated. The parasitoid wasp *Asobara japonica* is an ideal insect to investigate this because symbiotic and aposymbiotic strains are available: *Wolbachia*-infected Tokyo (TK) and noninfected Iriomote (IR) strains originally collected on the main island and southwest islands of Japan, respectively. We compared the oviposition behaviors of the two strains and found that TK strain females parasitized *Drosophila melanogaster* larvae more actively than the IR strain, especially during the first two days after eclosion. Removing *Wolbachia* from the TK strain wasps by treatment with tetracycline or rifampicin decreased their parasitism activity to the level of the IR strain. Morphological and behavioral analyses of both strain wasps showed that *Wolbachia* endosymbionts do not affect development of the host female reproductive tract and eggs, but do enhance host-searching ability of female wasps. These results suggest the possibility that *Wolbachia* endosymbionts may promote their diffusion and persistence in the host *A*. *japonica* population not only at least partly by parthenogenesis but also by enhancement of oviposition frequency of the host females.

## Introduction

Many parasitoid wasps harbor endosymbiotic bacteria *Wolbachia*, a single bacterial lineage in the alpha-group of the Proteobacteria. *Wolbachia* is generally a facultative reproductive parasite in arthropods, and invades the host population by reproductive manipulations such as cytoplasmic incompatibility, male killing, feminization, or thelytokous parthenogenesis [1–5]. *Wolbachia*-induced parthenogenesis is most commonly found in haplodiploid organisms, such as Hymenoptera [6–8]. In uninfected haplodiploid organisms, fertilized eggs develop into diploid daughters and unfertilized eggs develop into haploid sons, while infection with *Wolbachia* causes diploidization of the haploid eggs by alteration of meiotic and/or mitotic processes, resulting in the production of daughters from unfertilized eggs [9–11]. Since an asexual female produces only daughters, all of her offspring will contribute to the next generation. Therefore, an asexual population can grow faster than a sexual population and when they are in competition, the asexual population should outcompete the sexual one [12].

Aside from such reproductive effects of *Wolbachia* infection, recent findings in *Diptera* indicate that *Wolbachia* may also modify the host’s physiologies such as metabolism and immunity. In *D*. *melanogaster*, *Wolbachia* may play a role as a nutritional mutualist by affecting iron utilization by the hosts [13,14]. *Wolbachia* infection has also been shown to generate oxidative stress in one *Aedes aegypti* cell line, which reacts by the overexpression of host antioxidant genes [15]. This finding must be closely related with the fact that reactive oxygen species (ROS) are known to play a major role in immune response to pathogens [16,17]. Furthermore, *Wolbachia* is known to induce resistance against RNA viral infection in *D*. *melanogaster* and *D*. *simulans* [18,19], and against several arboviruses including dengue, yellow fever, and Chikungunya viruses in the mosquito *A*. *aegypti*, by priming the innate immune system [20–22].

Although increasing evidence is emerging on the phenotypic effects of *Wolbachia* infection on host reproduction and physiologies in non-Hymenopteran species, it is unknown whether it exerts similar effects on the physiologies of parasitoid wasps. Furthermore, it remains ambiguous whether *Wolbachia* causes other phenotypic effects such as behaviors of host insects, parasitoid wasps, in a particular symbiotic association in which *Wolbachia* affects parasitism processes through its effect on wasp oviposition. The objective of this study was to clarify the effect of *Wolbachia* on behavioral traits of the host female wasps. Here, we compared symbiotic and aposymbiotic parasitoid wasps in terms of parasitism activities of females using *Asobara japonica* because *Wolbachia*-infected Tokyo (TK) and noninfected Iriomote (IR) strains, which are originally collected on the main and southwest islands of Japan, respectively, are available [23–26]. Behavioral analyses demonstrated that symbiotic TK strain females parasitize *D*. *melanogaster* larvae significantly more actively than the aposymbiotic IR strain especially during an early adult stage. A series of analysis revealed that *Wolbachia*-induced parasitism activity of female wasps is primarily due to the enhancement of their host-searching ability

## Materials and Methods

### Animals

*Wolbachia*-infected (Tokyo, TK) and uninfected (Iriomote, IR) strains of *Asobara japonica* were originally collected from Tokyo and Iriomote-jima island, respectively　[27]. These strains were kindly provided by M.T. Kimura (Hokkaido University) and subsequently maintained under laboratory conditions as follows. Each generation, 5–10 female wasps were allowed to parasitize about 200–300 larvae of *D*. *similans* in glass vials (35 x 200 mm) at 23°C under 16L:8D regime. For experiments, *yellow-white* (*y w*) strain of *D*. *melanogaster* was used as a host [23,24].

### Antibiotic treatment

Removal of *Wolbachia* was performed basically according to the procedure of Dedeine et al. [28] as follows. Parasitoid eggs and larvae were exposed to tetracycline or rifampicin through hemolymph of the hosts which had been fed *Drosophila* medium containing 2 mg/ml tetracycline or 1 mg/ml rifampicin for 24 h. After pupariation occurred, host pupae were taken into a different vial and wasps emerged from hosts were collected. Removal of *Wolbachia* was confirmed by PCR analysis as follows.

### PCR, RT-PCR, and quantitative real-time PCR

PCR analysis was conducted using genome DNA samples prepared from whole bodies of *A*. *japonica* wasps according to the previously described procedure [29]. RT-PCR was conducted essentially according to the previously described procedure [30] as follows. Total RNAs were prepared from test tissues of parasitoid wasps using TriPure Isolation reagent (Roche Applied Science, USA). First-strand cDNA was synthesized with oligo(dT)_12-18_ primer using ReverTra Ace RT-PCR kit (Toyobo, Japan) according to the manufacturer’s protocol. The cDNAs for target genes were amplified with a specific primer pair indicated in Table 1. PCR was conducted under the following conditions: 25~35 cycles at 95°C for 30 s, 52~55°C for 30 s, and 72°C for 45 s.

**Table 1:** List of PCR primers used in this study.

***rp49***	
FW	AAAGGTATYGAYAACAGAGT
RV	TATTCSTTCYCCYCARATCG
***Wsp***	
FW	TGGTCCAATAAGTGATGAAGAAAC
RV	AAAAATTAAACGCTACTCCA
***WP1***	
FW	TTGTAGCCTGCTATGGTATAACT
RV	GAATAGGTATGATTTTCATGT
***Orco***	
FW	AAYAATCCKAACAGCCATCC
RV	CTGTTKGGMACKACGGTGTC
Common RACE Primers	
***Qt***	CCAGTGAGCAGATGACGAGGACTCGAGCTCAAGCTTTTTTTTTTTTTTTTTV
***Qo***	CCAGTGAGCAGAGTGACG
***Qi***	GAGGACTCGAGCTCAAGC
Primers for 5'-RACE	
***Aj-Orco-R1***	AAGAGCAGCCACGAGCAGAAGAGAACATCG
***Aj-Orco-R2***	TGATTCCGGTCCAGCAAAGT
Primers for 3'-RACE	
***Aj-Orco-F1***	CCATCACCAATGAAACGTCT
***Aj-Orco-F2***	GGTTTTCCAATTCTACTGGC

Quantitative real-time PCR was carried out with the cDNAs in a 20 ml reaction volume of LightCycler Fast DNA Master SYBR Green I (Roche Applied Science, USA) using the Light-Cycler 1.2 instrument and software (Roche Applied Science) [31]. The PCR cycling conditions were denaturation at 95°C for 10 min, followed by 45 cycles of heating at 95°C for 10 s, annealing at 55°C for 5 s, and extension at 72°C for 15 s. Using the second derivative maximum method provided in the LightCycler software (version 3.5), a standard curve was generated by plotting the external standard concentration against threshold cycle. The software automatically calculated PCR product concentration for each tissue sample. All samples were analyzed in duplicate, and assay variation was typically within 10%. Data were normalized according to the expression level of *rp49* determined in duplicate by reference to a serial dilution calibration curve.

### Activity of parasitism

To measure parasitism activity of test wasp females, one female of TK (or antibiotic-treated TK strains) or one pair of IR strain was put in a 30 ml glass vial with *Drosophila* medium containing host *D*. *melanogaster* larvae. IR strain females can mate within a few hours after eclosion. Therefore, we used only mated IR females (even day 0) in all experiments to eliminate a possible effect of mating on parasitism behavior. Furthermore, we have also demonstrated that the presence of males did not cause any change of oviposition rates of IR strain wasps. Detailed conditions were indicated in each experiment.

### Y-tube olfactory assay

The olfactory responses of *A*. *japonica* female wasps to odors were tested in a Y-tube olfactometer. The olfactometer consisted of a glass Y-tube (base 4.5 cm long; Y-arms each 4.5 cm long; 10 mm inner diameter). The Y-tube apparatus was modified after the design of Carroll et al. [32]. The base of the Y-tube was connected to a Teflon tube of similar size that was attached directly to the vacuum source. Odor sources, 50 μg of *Drosophila* medium with or without *D*. *melanogaster* larvae, were placed at either end of the Y-arms and the odors were extracted through the base arm at a flow rate of 2L/min by a vacuum pump to ensure a steady flow. Ice-anesthetized test wasps were introduced individually by disconnecting the Y-tube at its base and allowing the wasp to move into the olfactometer. After wasp recovered from anesthesia, the tube was reconnected to reestablish the airflow from the odor sources through the arms and out at the base towards the vacuum pump. A choice and consumed time were recorded when a test wasp reached either end of Y-arms. Test wasps were recorded as ‘not moved’ when they remained in the base arm. Each test was terminated after 30 min from the initiation of airflow. To avoid positional bias, odor chambers were switched after every replicate.

### Morphological analysis of lateral oviduct

Paired lateral oviducts were dissected from *A*. *japonica* female wasps and the magnification images were taken with a microscope with camera attachment. Sizes (length and width) of the tissues were measured using Image J National Institute of Health, USA). The number of eggs harbored in the oviducts was also counted under a microscope.

### Sequencing of *A*. *japonica Orco* gene

RT-PCR was carried out using cDNA prepared from antennae of day 0 females and degenerate primers designed from conserved sequences of *Apis mellifera* odorant receptor 2 (Or2), transcript variant X1, mRNA (gi 571501583) and *Microplitis demolitor* odorant receptor coreceptor (LOC103571567), mRNA (gi 665785588). Amplified fragments *of A*. *japonica Orco gene* were sequenced by using BigDye Terminator v3.1 (Applied Biosystems, USA) with 3130 Genetic Analyzer (Applied Biosystems, USA) [33]. Alignment of sequences was carried out on BioEdit Sequence Alignment Editor 7. 0. 9. 0. (Ibis Biosciences, USA).

To obtain full-length cDNA, 5’ rapid amplification of cDNA ends (5’RACE) was performed using 5’RACE kit (Invitrogen, Carlsbad, CA) as described previously [30]. All identified cDNA sequences were subjected to computer-assisted sequence analysis using GENETYX-MAC Ver. 13.1.7 (Software Development Co., Tokyo, Japan).

### Statistical analyses

For comparison of parasitism activity, development of ovary and preference for host odor, Tukey’s HSD tests were carried out. As a result of normality test, Shapiro-Wilk test, it was found that each data set does not deviate from the normality. All statistical analyses were performed using JMP 9.0.2 (SAS Institute).

## Results

### Frequencies of parasitism by TK and IR strains

**Fig 1:**
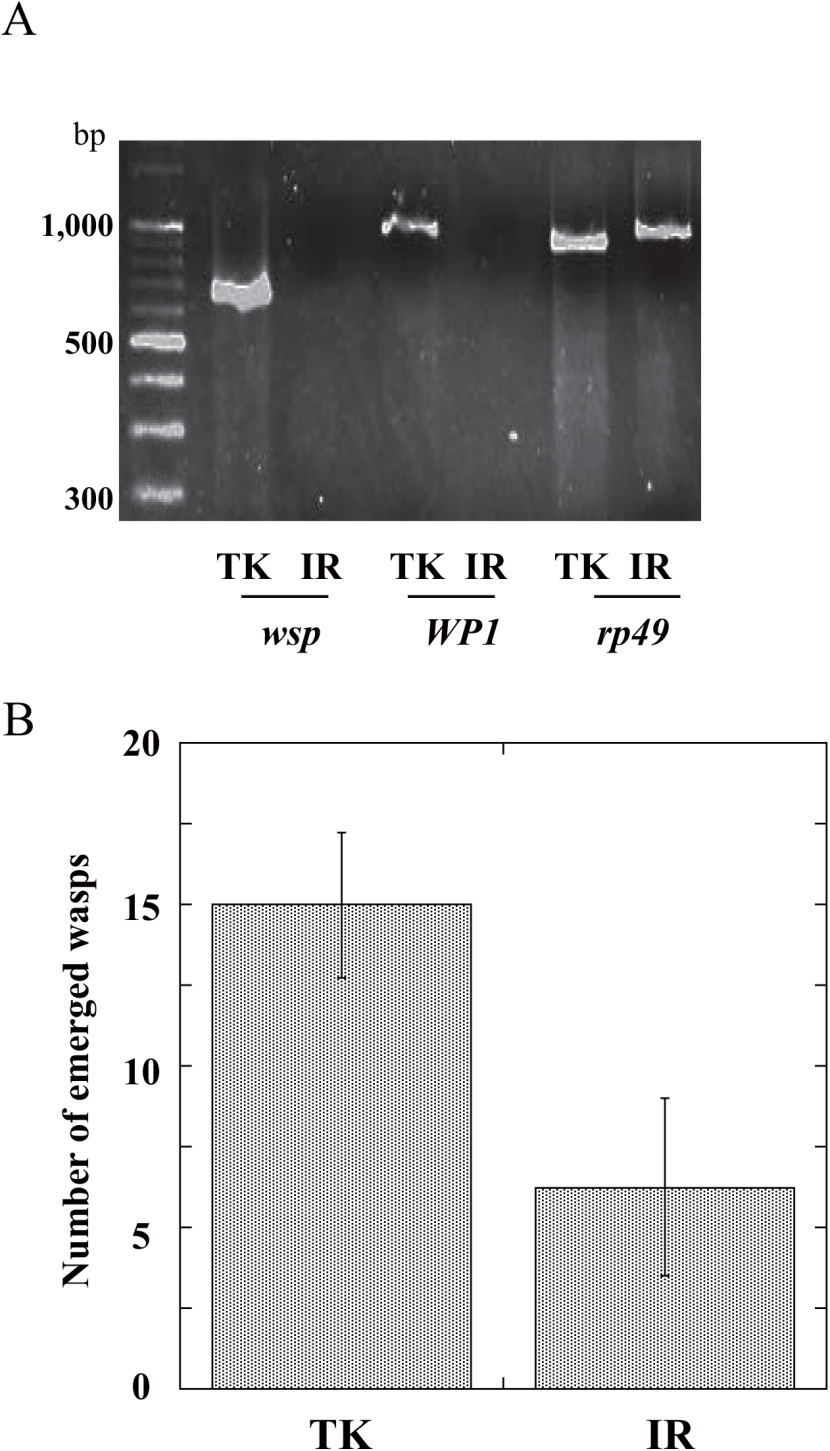
*(A)* RT-PCR of *Wolbachia* specific genes, *Wolbachia* surface protein gene (*wsp*) and *Wolbachia* protein 1 gene (*WP1*), and *A*. *japonica* ribosomal protein 49 gene (*rp49*) in whole bodies of *A*. *japonica* wasps. *(B)* The number of offspring wasps emerged from parasitized female. One female wasp randomly selected from day 0 to day 2 adults of TK or IR strain colony was put in a glass vials (35 x 120 mm) containing 200–300 *D*. *melanogaster* larvae and allowed to freely parasitize for 15 h. In the case of IR strain, mated females were used. Each value represents the mean ± S.D. for 16 independent determinations. Ten wasps of each strain were used for each determination. P = 0.056 (Tukey’s HSD).

We first confirmed that the TK strain flies harbored *Wolbachia pipientis* but the IR strain did not by PCR with two primer sets specific to *W*. *pipientis* genes (Fig 1A). To roughly compare parasitism activity by both strains, one young female of each strain was put into a 30 ml glass tube containing a *D*. *melanogaster* colony with *Drosophila* medium. All wasps were removed 15 h later, and *D*. *melanogaster* colonies were maintained until all wasp offspring emerged from the hosts. The number of the TK strain offspring was higher than those of the IR strain (Fig 1B). Although we did not count the number of eggs oviposited by both strain females, these results were interpreted to imply a possibility that TK strain female wasps with *Wolbachia* possess higher parasitic activity than IR strain animals.

### Effect of *Wolbachia* on oviposition rates of host wasps

**Fig 2:**
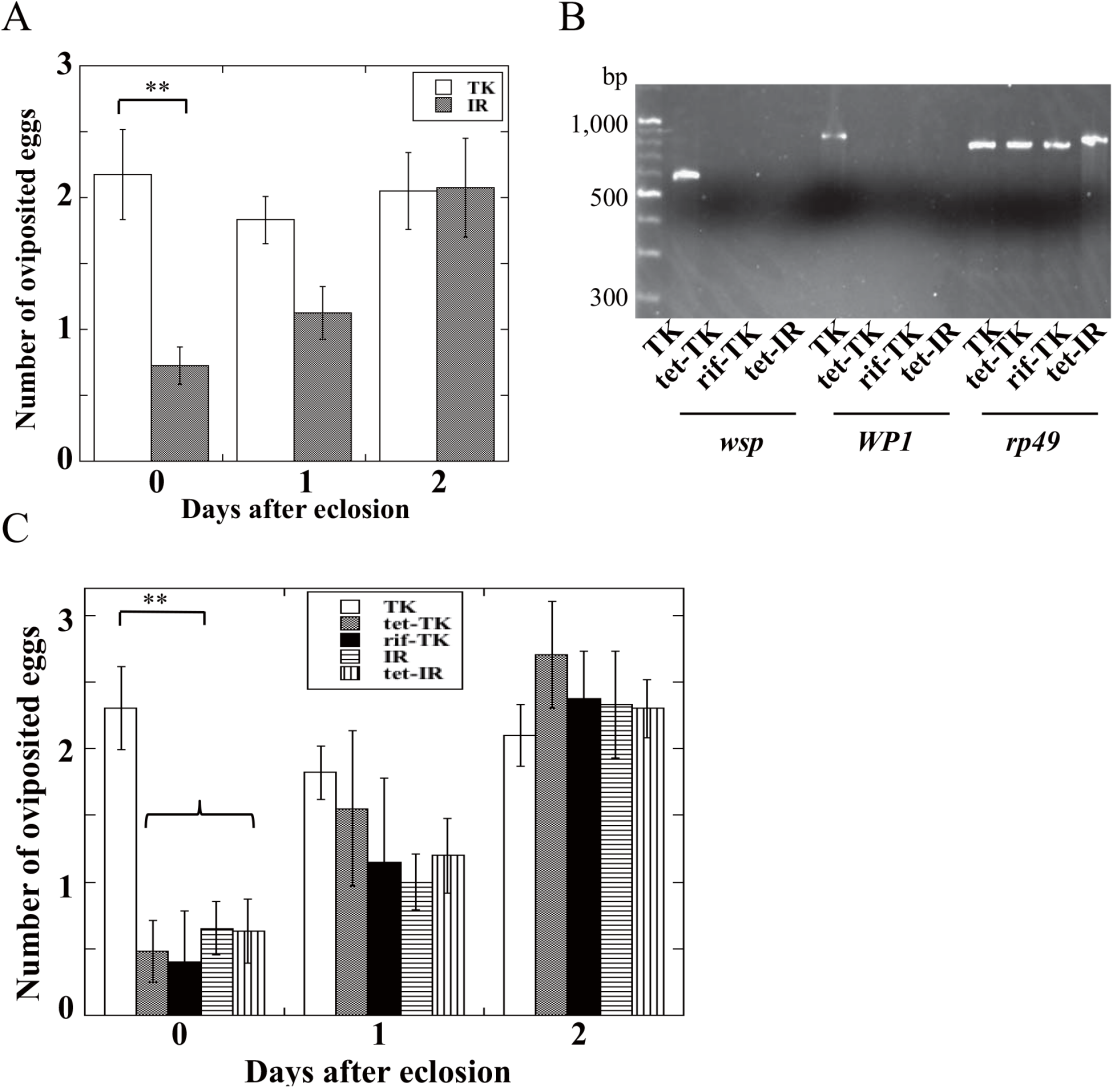
*(A)* The number of eggs oviposited in one host larva by TK or IR strain wasp during first 3 days after eclosion. Each value represents the mean ± S.D. for 18 independent determinations. Significant difference indicated by Tukey’s HSD (**P<0.01). One female wasp of each strain was allowed to parasitize for 24 h during each determination. In the case of IR strain, mated females were used. *(B)* Expression of *wsp*, *WP1*, and *rp49* in tetracycline-treated TK strain (tet-TK) wasps. *(C)* The number of eggs oviposited in one host larva by TK or IR strain wasp during first 3 days after eclosion. In this assay, we used only mated IR females and we verified that the presence of males did not cause any change of oviposition rates of IR female wasps. Each value represents the mean ± S.D. for 15 independent determinations. Significant difference indicated by Tukey’s HSD (**P<0.01). One female wasp of each strain was allowed to parasitize for 24 h during each determination.

To seek detailed differences in parasitism behavior between TK and IR strains, we counted the number of eggs in *D*. *melanogaster* larvae that had been oviposited by both strain females during first three days after eclosion (Fig 2A). On day 0, the number of eggs oviposited by TK strain females was 2~3 times higher than that of IR strains. The difference in oviposition rates between both strains decreased on day 1 and disappeared on day 2 (Fig 2A). These data suggested the possibility that *Wolbachia* enhances parasitism behavior especially during early adult stages of the wasp. To examine this, we eliminated *Wolbachia* by using *D*. *melanogaster* larvae fed an antibiotic (tetracycline (tet) or rifampicin (rif)) as the wasp’s hosts. This treatment succeeded in the elimination of *Wolbachia* from TK strain wasps (Fig 2B). TK stain female wasps without *Wolbachia* showed significantly lower oviposition rates on day 0 compared to the normal TK strain (Fig 2C). Furthermore, tet-treated IR strain wasps did not show any significant change in their oviposition rates, indicating that tet-treatment itself did not affect on the parasitism behavior of wasps (Fig 2C). Based on these results, it is reasonable to assume that the active parasitism in young adults of the TK strain wasps is due to the presence of symbiont *Wolbachia*.

### Effect of *Wolbachia* on development of host wasp reproductive tissues

**Fig 3:**
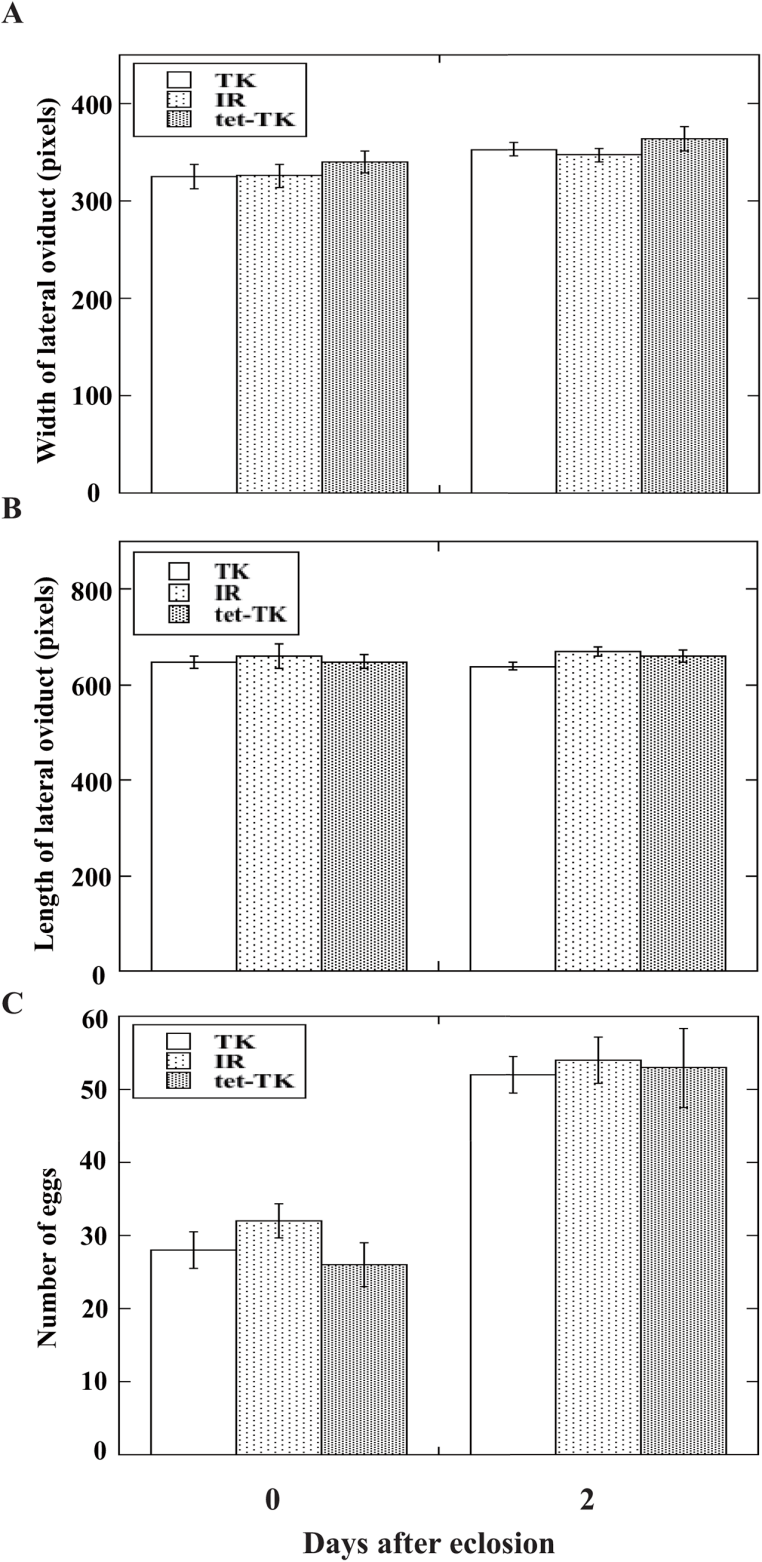
Width *(A)*, length *(B)*, and the number of eggs *(C)* in lateral oviducts of *A*. *japonica* TK, IR, and tet-TK strains. Each value represents the mean ± S.D. for 7–9 independent determinations. One female wasp of each strain was used for each determination.

To examine whether *Wolbachia* affects development of the reproductive tracts and eggs in female hosts, we dissected the lateral oviducts and measured their sizes. Neither the widths nor the lengths of TK, IR, and tet-treated TK (tet-TK) strains were different even on day 0 and day 2 of adult stages (Fig 3A and 3B). Furthermore, the number of eggs in the oviducts was not different among the three strains. Therefore, *Wolbachia* symbiosis did not affect reproductive maturation rates of the parasitoid wasps.

### Effect of *Wolbachia* on parasitism behavior of host wasps

**Fig 4:**
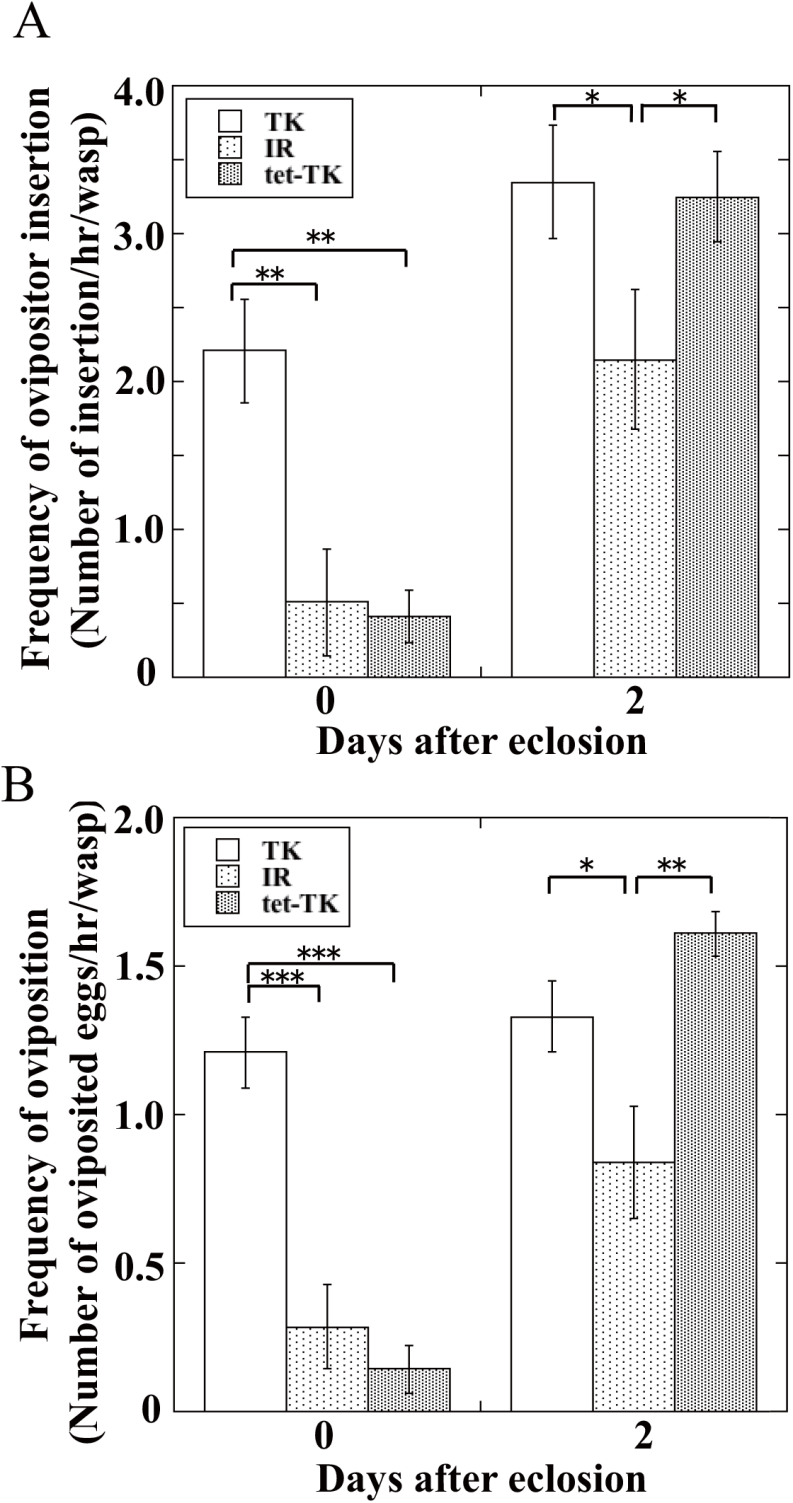
Frequencies of ovipositor insertion behavior *(A)* and oviposition by wasp *(B)* of *A*. *japonica* TK strain, IR strain, or tet-TK strain. In the case of IR strain, mated females were used. Each value represents the mean ± S.D. for 6–7 independent determinations. Significant difference indicated by Tukey’s HSD (*P<0.05, **P<0.01, ***P<0.001). Two female wasps of each strain were put in an assay plate containing twenty *D*. *melanogaster* larvae with their medium, and each test wasp was allowed to parasitize for 1 h during each determination.

To find the main cause of high oviposition rates of young TK strain female wasps, we compared behavioral traits of both strains of female wasps. First, we measured the frequency of ovipositor insertion into an artificial diet containing host larvae by female wasps of both strains. TK strain females inserted their ovipositors much more frequently than IR strain and tet-TK strain females did on day 0, but afterwards, the frequencies of ovipositor insertion of IR and tet-TK strains increased to a level almost equivalent to that of the TK strain on day 2 (Fig 4A). The number of eggs oviposited by the three strains of females also showed the same tendencies as the ovipositor insertion frequencies: the TK strain laid eggs much more actively than IR and tet-TK strains did on day 0, but the difference in oviposition activities between TK and other two strains was decreased on day 2 (Fig 4B).

**Fig 5:**
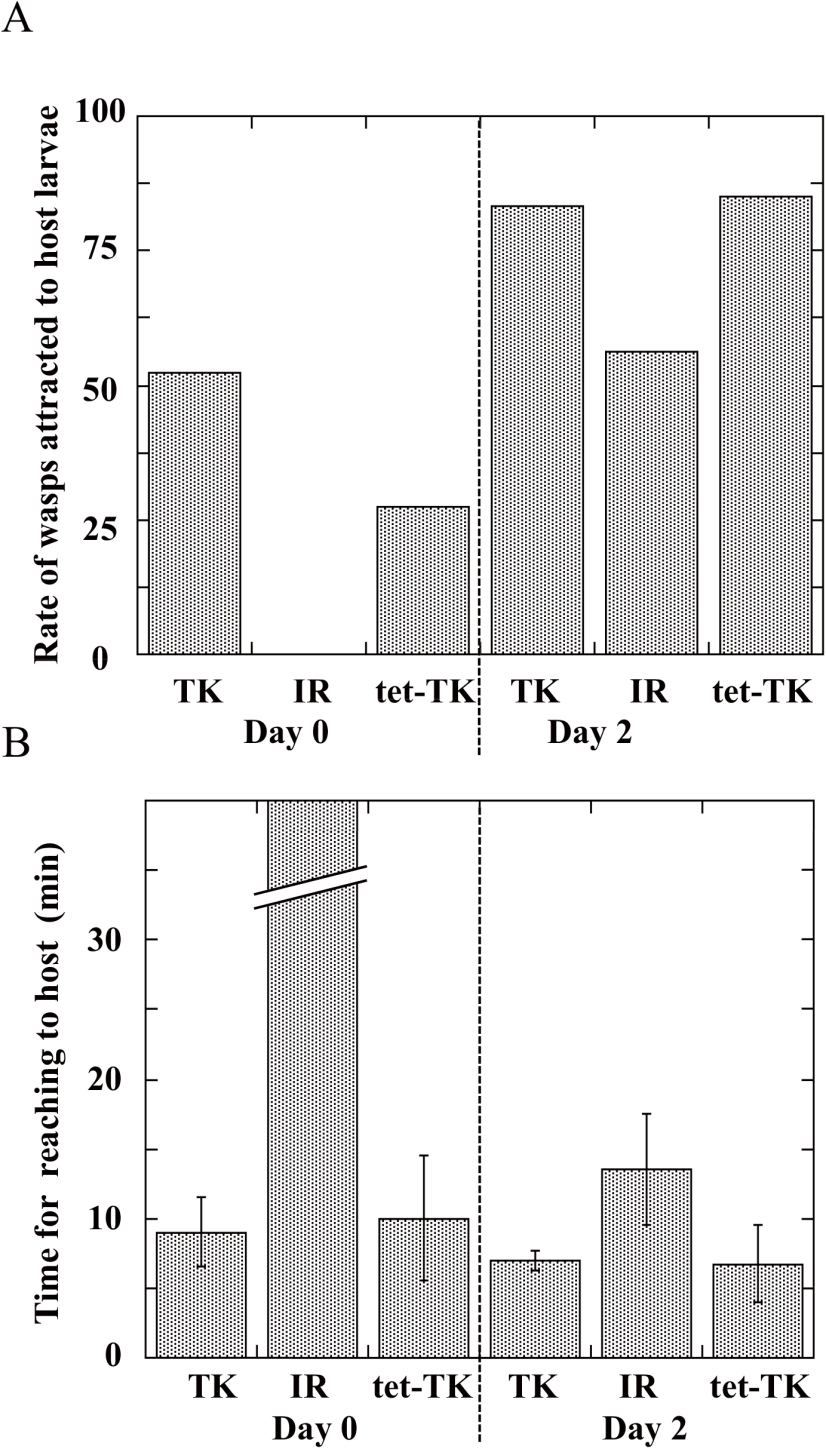
*(A)* Rate of *A*. *japonica* TK, IR, and tet-TK strain wasps attracted to *Drosophila* medium with *Drosophila* larvae. Each value represents the mean of tested wasps; 11 (day 0) and 21 (day 2) TK, 7 (day 0) and 9 (day 2) IR, and 7 (day 0) and 8 (day 2) tet-TK strains were used for the determination. In the case of IR strain, mated females were used. Although TK and IR strain wasp colonies were maintained using *D*. *similans* as a host, we have checked that switching the host from *D*. *similans* to *D*. *melanogaster* does not change in the behaviors of both strain wasps. *(B)* Time for wasps to reach *Drosophila* medium with *Drosophila* larvae. Each value represents the mean ± S.D. for above numbers of tested wasps. Mean time of IR strain wasps was significantly longer than those of TK and tet-TK strains on day 0 (P<0.001, Tukey’s HSD). Other explanations are as in (*A*).

Recognition of host *Drosophila* larvae by three strains of female wasps was determined in a Y-tube olfactometer. TK strain females were attracted to the smell of the host larvae much more strongly than IR and tet-TK strains were on day 0, but the behavioral differences between TK and other strains were reduced on day 2 (Fig 5A). Furthermore, TK strain females reached an artificial diet containing the host larvae much more quickly than IR and tet-TK strains did on day 0 (Fig 5B). The difference in host searching behavior between TK and IR strains was preliminarily demonstrated not to be changed when we had switched the host *Drosophila* species from *D*. *similans* to *D*. *melanogaster* for maintaining wasp colonies.

### Effect of *Wolbachia* on olfactory receptor gene expression in host wasps

**Fig 6:**
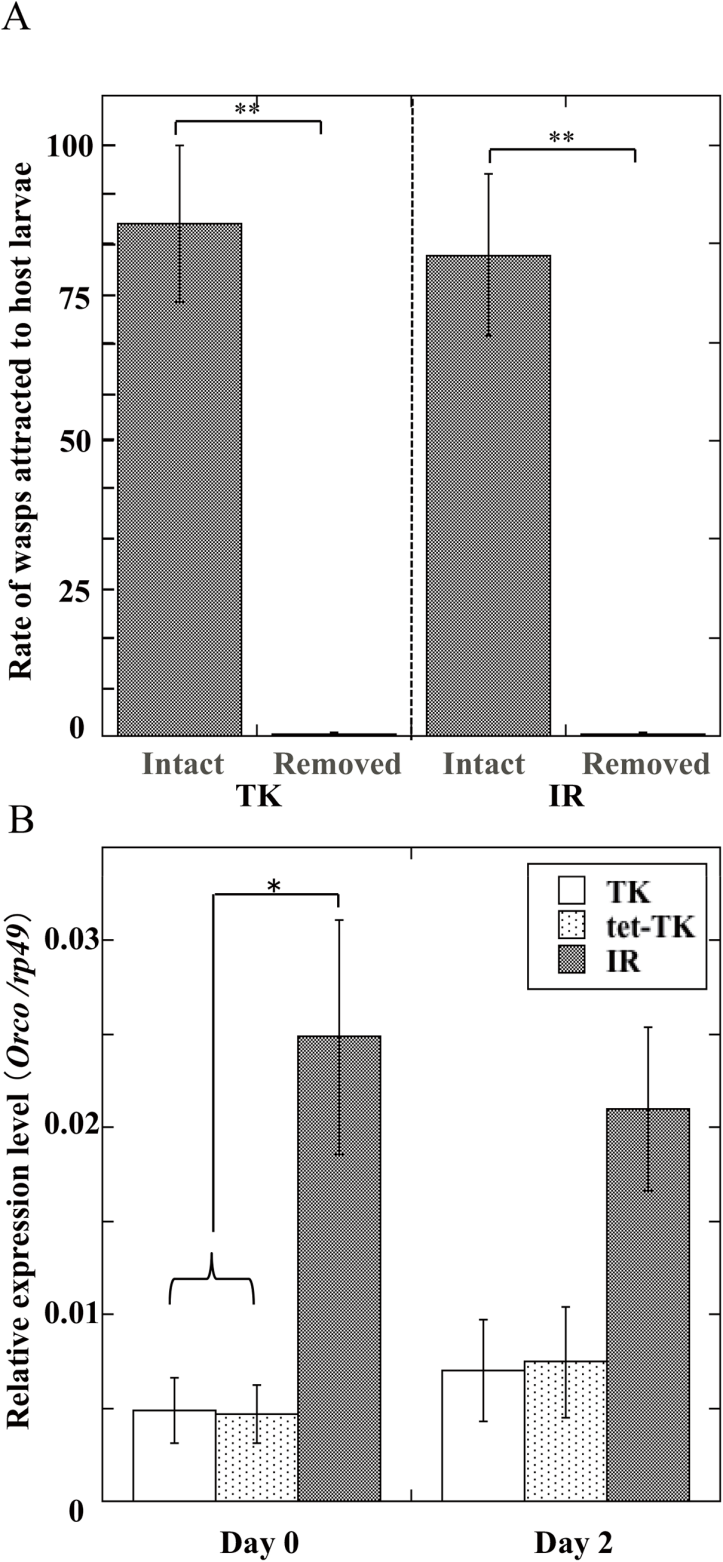
*(A)* Rate of *A*. *japonica* wasps reached to *Drosophila* medium with *Drosophila* larvae before and after removing their antennae. Each value represents the mean ± S.D. for 8 independent determinations. Significant difference indicated by Tukey’s HSD (*P<0.01, **P<0.001). Fifteen day 2 female wasps of each strain were used for each determination. In the case of IR strain, mated females were used. *(B)* Relative expression levels of *Orco* in antennae of *A*. *japonica* TK, tet-TK, and IR wasps on day 0 and day 2. Each value represents the mean ± S.D. for 5–8 independent determinations. Significant difference indicated by Tukey’s HSD (*P<0.05). Twenty wasps of each strain were used for each determination.

Finally, we tested whether the behavioral differences between TK and IR (or tet-TK) strains were due to a difference in olfactory abilities in these strains. Since the antennae have been reported to be the sensorial organs used by female parasitoid wasps to locate their hosts, we cut off the antennae of both strains and observed oviposition behaviors of female wasps. This treatment strongly destroyed their host searching ability and removed the difference in TK and IR strain females, (Fig 6A).

In *Drosophila*, odorant receptor co-receptor (Orco, Or83b) is expressed in almost all olfactory receptor neurons and found to form heteromers with other odorant receptor proteins [34], indicating that Orco is essential for *Drosophila* to sense all odorants. Since it also has been demonstrated that other insect species, such as silkworm and honeybee, possess an Orco family protein [35], we made a degenerate *Orco* primer set in the conserved region of reported genes and sequenced by RT-PCR (S1 Fig). The full length of *Orco* genes was amplified by repeated 5’- and 3’-RACE reactions using cDNAs prepared from both strain wasp antennae. The homologies of the nucleotide sequences and the deduced amino acid sequences of both strain *Orco* genes were found to be 99.5% and 99.8%, respectively, indicating that the structures of *Orco* genes in both strains are almost identical (S2 and S3 Figs). We then performed quantitative real-time PCR by using cDNA expressed in the antennae. Expression levels of the *A*. *japonica Orco* orthologous gene were unexpectedly higher in IR than those in TK strains both on day 0 and day 2. Furthermore, the low gene expression in TK strain was not elevated by tet-treatment, indicating that the better host searching ability of TK strain cannot be simply explained by expression levels of *Orco* (Fig 6B).

## Discussion

All populations of *A*. *japonica* collected on the main islands of Japan have been reported to harbor *Wolbachia*. Although uninfected populations were exceptionally found on the small southern islands, the total infection of the main island populations suggests that *Wolbachia* infection has an ecological advantage for this wasp species to extend its habitat. *Wolbachia-*induced parthenogenesis is most generally thought to be beneficial for host insects to increase their population; in the case of haplodiploid organisms such as wasps, diploidization of the haploid eggs caused by *Wolbachia* produces daughters from unfertilized eggs [36]. Such a reproductive manipulation allows *Wolbachia* to increase infected wasp populations, which increases their transmission [37].

In this study, we sought additional positive effects of *Wolbachia* infection for *A*. *japonica* wasps other than the reproductive manipulation, and found that the oviposition rates of *Wolbachia-*infected (TK strain) females were clearly higher than those of uninfected (IR strain) females on day 0 of the adult stage. Therefore, *Wolbachia* infection enhances the oviposition behavior of *A*. *japonica* wasps during an early adult stage. This is certainly beneficial for the *Wolbachia-*infected stain because life spans of both strains are not significantly different in glass culture tubes containing host larvae and their artificial diet: life spans of TK and IR strains were calculated to be 4.33 ± 1.22 days (n = 9) and 4.60 ± 2.22 (n = 10), respectively, by our preliminary experiments. Furthermore, we observed that *A*. *japonica* females of TK strain could parasitize soon after the emergence from the host in the field as well as in the laboratory. Given that their average life span must be shorter under natural conditions than in the culture tube due to the presence of predators, infection, and various physical accidents, it is reasonable to think that this behavioral trait must become more beneficial for wasps under natural conditions. To reveal the mechanism underlying the elevation of the oviposition rates of young *Wolbachia-*infected females, we compared the sizes of the reproductive tracts and number of eggs in the oviducts of TK strain and IR strain young wasps. Neither the size of the lateral oviduct nor the number of eggs was different between both strains of wasps. However, we found that the oviposition activity of the TK strain was significantly higher than that of the IR strain during the young adult stage: the numbers of oviposited eggs in host larvae, as well as frequency of ovipositor insertion to the artificial diet containing host larvae, were significantly higher in the TK strain compared to the IR strain on day 0. This difference was demonstrated to be primarily due to *Wolbachia-*mediated enhancement of host-searching ability of the TK strain. The better host searching ability of TK strain was verified irrespective of host *Drosophila* species: switching the host from *D*. *melanogaster* to *D*. *similans* did not change the behavioral difference between TK and IR strain wasps (data not shown).

It has been reported that the sensorial organs used by female parasitoid wasps to locate and evaluate their hosts are mostly present on their antennae where olfactory chemosensory sensilla have been described. Many olfactory receptors are present in the sensilla of antennae. Although they are highly diverse in insect species, the *Orco* family is exceptional: members share a highly conserved gene sequence among different species and play an important role in regulating insect behavior [38]. Therefore, we examined whether *Wolbachia* infection affects expression of *A*. *japonica Orco*. The expression levels of the ortholog of the *Drosophila Orco* gene were unexpectedly higher in IR than in TK strain wasps, suggesting at least that the high host-searching ability of TK strain is not due to a high expression level of *Orco* under the effect of *Wolbachia* infection. Although it is known that *Orco* plays an essential general roles in olfaction of *Drosophila* [39], there has not been reported the precise relationship between *Orco* expression levels and olfactory sensitivities. The present observation that tet-treatment of TK strain did not elevate the *Orco* expression level negated one possibility that extra-expression of *Orco* negatively affects olfaction or expression level of *Orco* does not affect any substantial olfactory ability. However, this result is not surprising because it is reasonable to consider that host-searching as well as oviposition behaviors cannot be controlled in a simple way by a sole gene. The fact that cutting off the antennae of both strain females drastically decreased their host searching abilities (Fig 6A) could be explained by postulating that both strain females cannot normally move or act without olfactory stimuli through the sensorial organ. Further investigation is required to clarify the relationship between expression levels of olfactory receptor genes such as *Orco* in the antenna and the olfactory sensitivities.

Pannebakker et al. [40] recently compared transcriptomes of the resting and ovipositing female parasitoid *Nasonia vitripennis* using a “DeepSAGE” gene expression approach. They identified 332 tags that were significantly differentially expressed between the two treatments, and found that nine of the most abundant differentially expressed tags showed greater expression in ovipositing females, including the genes *purity-of essence* (*poe*) (associated with behavioral phenotypes in *Drosophila melanogaster*) and *glucose dehydrogenase*. The *poe* protein is an evolutionarily conserved, large membrane protein containing a motif that affects behavior and synaptic transmission of *D*. *melanogaster*. Since it has been reported that two mutants of *poe* in *Drosophila* cause both increased nervous excitability and reduced motor function [41] and mutants also affect peripheral nerve morphology [42], it is reasonable to assume that *Wolbachia* infection might cause enhancement of such transcription factors in the *A*. *japonica* female wasp, resulting in the change of their oviposition behavior. Furthermore, approximately three-quarters of the changes involve greater expression in resting females, and enrichment analysis suggests that these down-regulated genes are more likely to be involved in various metabolic processes than expected by chance, suggesting that as female wasps move from resting to ovipositing, aspects of their metabolism are down-regulated, focusing gene expression on other processes [40]. These results can be interpreted to indicate that oviposition behavior is associated with changes in a variety of gene expressions in female whole bodies. Another recent comparative analysis of gene expression in ovaries between *Wolbachia-*infected and the uninfected parasitoid wasp *Asobara tabida*, a close species to *A*. *japonica*, showed that *Wolbachia* might interfere with numerous biological processes such as oogenesis, programmed cell death, and immunity [43]. These reports together with our unexpected result on *A*. *japonica Orco* expression imply that *Wolbachia-*induced enhancement of host-searching ability in the TK strain is not driven by a change of a single olfactory receptor gene that directly controls sensing of the host smell.

## Supporting Information

S1 Fig Comparison of nucleotide sequences of 5 insect species odorant receptor coreceptor genes (*Orco*). The fragment of *Orco* cDNA was prepared from antennae of *A*. *japonica* female wasps. The base sequence was analyzed as described in Materials and Methods.
(PDF)

S2 Fig Comparison of nucleotide sequences of TK and IR strain odorant receptor coreceptor genes (*Orco*). Whole *Orco* cDNAs were prepared from antennae of both strain female wasps by RT-PCR. Start and stop codons were red-colored.
(PDF)

S3 Fig Comparison of deduced amino acid sequences of TK and IR strain odorant receptor coreceptor genes (*Orco*). Whole *Orco* cDNAs were prepared from antennae of both strain female wasps by RT-PCR. Only one amino acid, Lue^402^, is replaced with Phe.
(PDF)
