# Supplementary figures and images for: Bacteria Endosymbiont, Wolbachia, Promotes Parasitism of Parasitoid Wasp Asobara japonica
Source: PLoS One. 2015 Oct 22;10(10):e0140914. doi: 10.1371/journal.pone.0140914 (PMC4619603; doi:10.1371/journal.pone.0140914)

S1 Fig.

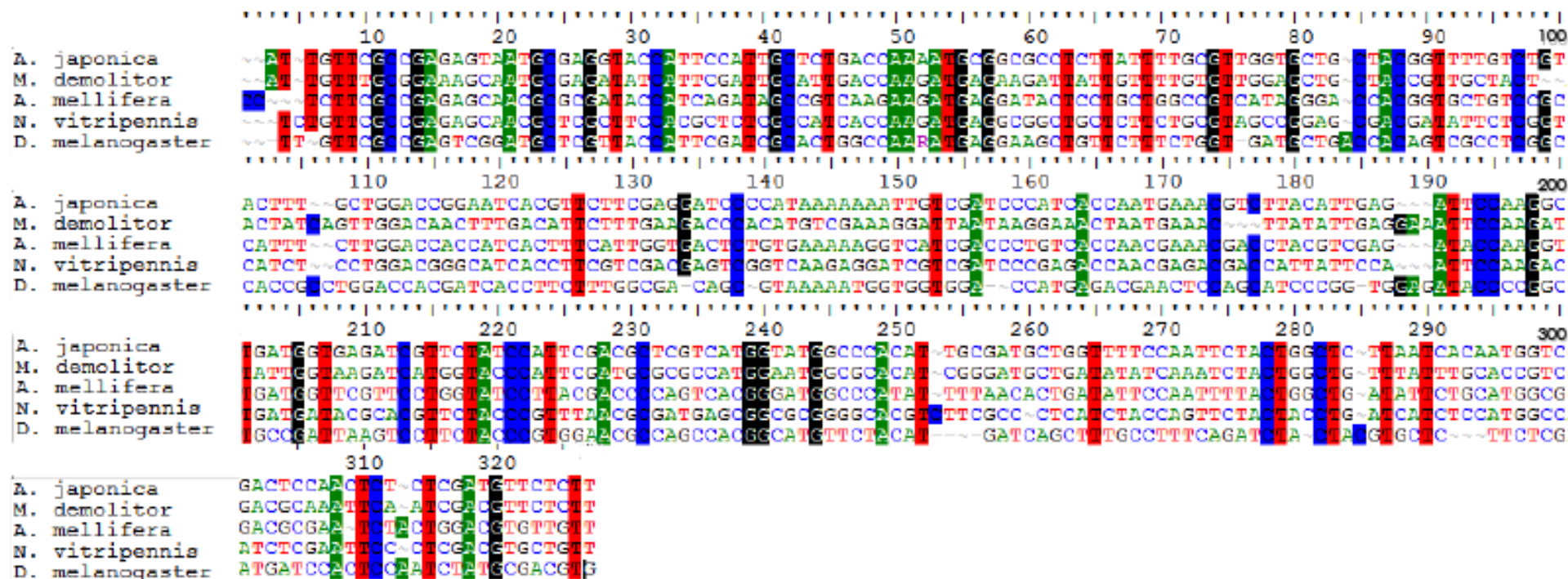

Supplement: S1 Fig — The fragment of Orco cDNA was prepared from antennae of A. japonica female wasps. The base sequence was analyzed as described in Materials and Methods. (PDF) [file pone.0140914.s001.pdf]
