# Supplementary material for: Bacteria Endosymbiont, Wolbachia, Promotes Parasitism of Parasitoid Wasp Asobara japonica
Source: PLoS One. 2015 Oct 22;10(10):e0140914. doi: 10.1371/journal.pone.0140914 (PMC4619603; doi:10.1371/journal.pone.0140914)
Supplement: S2 Fig — Whole Orco cDNAs were prepared from antennae of both strain female wasps by RT-PCR. Start and stop codons were red-colored. (PDF) [file pone.0140914.s002.pdf]

S2 Fig.

```
Tokyo    GGAGATACAACGGCTTCAAGATGATGAAGACCAAGCATCAGGGCCTTGTGGCCGATCTCATGCCCAACATAAGGCTCATGCAAATCAGTGGACACTTTATGTTCAATTATTATGGGGAGG 120
Iriomote •••••••••••••••••••••••••••••••••••••••••••••••••••••••••••••••••••••••••••••••••••••••••••••••••••••••••••••••••••••••••• 120′

Tokyo    GCAAGAAGCTCATGCACAAAGTCTACTGCAGTGTCCACCTATTCCTGATAGTCCTTCAATTCGCGTTATGCGGAATTAACTTGGCAATGGAGAGTGGAGATGTTGATGATCTAACCGCCA 240
Iriomote •••••••••••••••••••••••••••••••••••••••••••••••••••••••••••••••••••••••••••••••••••••••••••••••••••••••••••••••••••••••••• 240′

Tokyo    ACACCATCACAGTACTTTTCTTCTTGCATCCCGTCGTGAAAGTGGTTTACTTTGCAATCAGAAGTAAATTATTCTACCGCACACTAGCCATTTGGAATAATCCCAATAGTCATCCATTGT 360
Iriomote •••••••••••••••••••••••••••••••••••••••••••••••••••••••••••••••••••••••••••••••••••••••••••••••••••••••••••••••••••••••••• 360′

Tokyo    TCGCCGAGAGTAATGCGAGGTACCATTCCATTGCTCTGACCAAAATGCGGCGCCTCTTATTTTGCGTTGGTGCTGCTACGGTTTTGTCTGTACTTTGCTGGACCGGAATCACGTTCTTCG 480
Iriomote •••••••••••••••••••••••••••••••••••••••••••••••••••••••••••••••••••••••••••••••••••••••••••••••••••••••••••••••••••••••••• 480′

Tokyo    AGGATCCCCATAAAAAAATTGTCGATCCCATCACCAATGAAACGTCTTACATTGAGATTCCAAGGCTGATGGTGAGATCGTTCTATCCATTCGACGCTCGTCATGGTATGGCCCACATTG 600
Iriomote •••••••G•••••••••••••••••••••••••••••••••••••••••••••••••••••••••••••••••••••••••••••••••••••••••••••••••••••••••••••••• 600

Tokyo    CGATGCTGGTTTTCCAATTCTACTGGCTCTTAATCACAATGGTCGACTCCAACTCTCTCGATGTTCTCTTCTGCTCGTGGCTGCTCTTCGCTTGCGAACAACTCCAGCATCTCAAGGCCA 720
Iriomote •••••••••••••••••••••••••••••••••••••••••••••••••••••••••••••••••••••••••••••••••••••••••••••••••••••••••••••••••••••••••• 720′

Tokyo    TCATGAAACCTCTTATGGAGCTAAGCGCAACCCTGGATACCGTAGTGCCCAACAGTAGTGAACTATTTAAGGCTGGAAGTGCCGATCATTTACGAGACACCAATGGCACTCAACCCCCAG 840
Iriomote •••••••••••••••••••••••••••••••••••••••••••••••••••••••••••••••••••••••••••••••••••••••••••••••••••••••••••••••••••••••••• 840′

Tokyo    CAACTCCCCAACAAGGGGATAATATGCTTGACTTGGATCTCCGGGGGATCTACAGTAATCGCCAGGATTTCACAGCGACATTCAGACAAACAGTTGGGCAATTCAATGGAGGAGTTGGAC 960
Iriomote •••••••••••••••••••••••••••••••••••••••••••••••••••••••••••••••••••••••••••••••••••••••••••••••••••••••••••••••••••••••••• 960′

Tokyo    CCAATGGGCTCACCAAGAAACAGGAGATGCTTGTAAGGAGTGCGATCAAGTATTGGGTCGAACGACACAAGCACGTGGTGAGACTGGTGACCGCTATTGGAGATGCTTACGGTGTTGCTC 1080
Iriomote •••••••••••••••••••••••••••••••••••••••••••••••••••••••••••••••••••••••••••••••••••••••••••••••••••••••••••••••••••••••••• 1080′

Tokyo    TTCTATTCCACATGCTTATCACCACAATCACCCTGACCCTACTCGCTTACCAAGCCACAAAGGTCAATGGCGTCAATGTTTATGCTGCAACGACAATTGGCTATTTGCTGTATTCCCTGG 1200
Iriomote •••••••••••••••••••••••••••••••••••••••••••••••••••••••••••••••••••••C•••••••••••••••••••••••••••••••••• 1200′

Tokyo    GGCAGGTCTTCTTGTTCTGTATACTTGGAAATCGTTTGATTGAAGAAAGTTCATCGGTGATGGAAGCAGCCTACTCTTGTCACTGGTATGACGGCTCCGAGGAGGCTAAAACATTTGTCC 1320
Iriomote •••••••••••••••••••C•••T•••••••••••••••••••••••••••••••••••••••••••••••••••••••••••••••••••••••••••••••••C•••• 1320′

Tokyo    AAATTGTGTGTCAGCAGTGTCAAAAAGCCATGTCTATATCAGGCGCTAAATTTTTCACGGTCTCTTTAGATCTCTTCGCGTCGGTTCTCGGCGCTGTTGTAACGTACTTCATGGTTTTGG 1440
Iriomote •••••••••••••••••••••••••••••••••••••••••••••••••••••••••••••••••••••••••••••••••G•••••••••••••••••••••• 1440′

Tokyo    TGCAGCTGAAATAGATTATCGGCGTTATGAAGGGTCATTGGAAGAGTTTCGAGGTGGAAATCAATAGAAGAACGTGGAAAATATGTGGCAAAGGGGATGAAAACAATTCACTTCCTTCAA 1560
Iriomote •••••••••••••••••••••••••••••••••••••••••••••••••••••••••••••••••••••••••••••••••••••••••••••••••••••••••••••••••••••••••• 1560′

Tokyo    CTTCAATTCTCCCATTGTATTTTTCGATTGTATCTTTCATTCTCAAGTGCCGCGGCACGCCATTTAAACTTATCCATTCGAGTCTTTAAGGATGTTTGTCCCTTCGGGTTGGGTGGGGGC 1680
Iriomote ••••••••••••••••••••••••••••••••••••••••••C••••••••••••••••••••••••••••••••••••••••••••••••••••••••••••••••A•••••••••••• 1680′

Tokyo    GATTCAATTTTTTCCCGCCCTTCTGAGGAAACCAAAATTTCAATACACCTGCTGCGCCCTTCAACCCCTGCCATAATAACTATATGTTCAAAATTATTATTAAAATTTTTCAATTAATCA 1800
Iriomote ••••••••••••••••••••••••••••••••••••••••••••••••••••••••T•••••••••••••••••••••••••••••••••••••••••••••••••••••••••••••T• 1800′

Tokyo    TTAAAAAAAAAAAAAAAAAA 1820
Iriomote •••••••••••••••••••• 1820′
```
