# Supplementary material for: Bacteria Endosymbiont, Wolbachia, Promotes Parasitism of Parasitoid Wasp Asobara japonica
Source: PLoS One. 2015 Oct 22;10(10):e0140914. doi: 10.1371/journal.pone.0140914 (PMC4619603; doi:10.1371/journal.pone.0140914)
Supplement: S3 Fig — Whole Orco cDNAs were prepared from antennae of both strain female wasps by RT-PCR. Only one amino acid, Lue402, is replaced with Phe. (PDF) [file pone.0140914.s003.pdf]

S3 Fig.

```
Tokyo    MMKTKHQGLVADLMPNIRLMQISGHFMFNYYGEGKKLMHKVYCSVHLFLIVLQFALCGIN  60
         ************************************************************
Iriomote MMKTKHQGLVADLMPNIRLMQISGHFMFNYYGEGKKLMHKVYCSVHLFLIVLQFALCGIN  60′

Tokyo    LAMESGDVDDLTANTITVLFFLHPVVKVVYFAIRSKLFYRTLAIWNNPNSHPLFAESNAR 120
         ************************************************************
Iriomote LAMESGDVDDLTANTITVLFFLHPVVKVVYFAIRSKLFYRTLAIWNNPNSHPLFAESNAR 120′

Tokyo    YHSIALTKMRRLLFCVGAATVLSVLCWTGITFFEDPHKKIVDPITNETSYIEIPRLMVRS 180
         ************************************************************
Iriomote YHSIALTKMRRLLFCVGAATVLSVLCWTGITFFEDPHKKIVDPITNETSYIEIPRLMVRS 180′

Tokyo    FYPFDARHGMAHIAMLVFQFYWLLITMVDSNSLDVLFCSWLLFACEQLQHLKAIMKPLME 240
         ************************************************************
Iriomote FYPFDARHGMAHIAMLVFQFYWLLITMVDSNSLDVLFCSWLLFACEQLQHLKAIMKPLME 240′

Tokyo    LSATLDTVVPNSSELFKAGSADHLRDTNGTQPPATPQQGDNMLDLDLRGIYSNRQDFTAT 300
         ************************************************************
Iriomote LSATLDTVVPNSSELFKAGSADHLRDTNGTQPPATPQQGDNMLDLDLRGIYSNRQDFTAT 300′

Tokyo    FRQTVGQFNGGVGPNGLTKKQEMLVRSAIKYWVERHKHVVRLVTAIGDAYGVALLFHMLI 360
         ************************************************************
Iriomote FRQTVGQFNGGVGPNGLTKKQEMLVRSAIKYWVERHKHVVRLVTAIGDAYGVALLFHMLI 360′

Tokyo    TTITLTLLAYQATKVNGVNVYAATTIGYLLYSLGQVFLFCILGNRLIEESSSVMEAAYSC 420
         *****************************************.******************
Iriomote TTITLTLLAYQATKVNGVNVYAATTIGYLLYSLGQVFLFCIFGNRLIEESSSVMEAAYSC 420′

Tokyo    HWYDGSEEAKTFVQIVCQQCQKAMSISGAKFFTVSLDLFASVLGAVVTYFMVLVQLK 477
         *********************************************************
Iriomote HWYDGSEEAKTFVQIVCQQCQKAMSISGAKFFTVSLDLFASVLGAVVTYFMVLVQLK 477′
```
